# Key Factors Determining the Self-Healing Ability of Cement-Based Composites with Mineral Additives

**DOI:** 10.3390/ma14154211

**Published:** 2021-07-28

**Authors:** Kamil Tomczak, Jacek Jakubowski, Łukasz Kotwica

**Affiliations:** 1 Department of Geomechanics, Civil Engineering and Geotechnics, AGH University of Science and Technology, 30-059 Krakow, Poland; ktomczak@agh.edu.pl; 2 Department of Building Materials Technology, AGH University of Science and Technology, 30-059 Krakow, Poland; lkotwica@agh.edu.pl

**Keywords:** self-healing, cementitious material, supplementary cementitious materials, mineral additives, fly ash, fluidized bed combustion, cracks in concrete, computer image processing, ultrasonic pulse velocity

## Abstract

This paper reveals the relationships between key factors that determine the ability of cementitious composites to self-heal autogenously and specific measures for quantifying the effects of this process. The following material factors: water-to-binder ratio (w/b), uniaxial compressive strength and age of the composite at the time of defect formation were considered, as well as the method and degree of damage to the tested material. The subjects of this study were mortars and concretes in which Portland cement was partially replaced, to varying degrees, with mechanically activated fluidized bed combustion fly ash (MAFBC fly ash) and siliceous fly ash. The samples were subjected to three-point bending or cyclic compression tests after 14 or 28 days of aging, in order to induce defects and then cured in water for 122 days. Microscopic (MO) and high-resolution scanning (HRS) observations along with computer image processing techniques were used to visualize and quantify the changes occurring in the macro-crack region near the outer surface of the material during the self-sealing process. Techniques based on the measurement of the ultrasonic pulse velocity (UPV) allowed the quantification of the changes occurring inside the damaged materials. Mechanical testing of the composites allowed quantification of the effects of the activity of the binder-supplementary cementitious materials (SCMs) systems. The analysis of the results indicates a significant influence of the initial crack width on the ability to completely close the cracks; however, there are repeated deviations from this rule and local variability of the self-sealing process. It has been shown that the compressive strength of a material is an important indicator of binder activity concerning crack width reduction due to self-sealing. Regardless of the crack induction method, the internal material changes caused by self-sealing are dependent on the degree of material damage.

## 1. Introduction

One of the concepts of increasing the durability of materials and structures made of these materials is self-healing. In this context, self-healing is understood as the ability of a material to spontaneously restore the original integrity of the structure, lost as a result of the formation of cracks and scratches, limiting the penetration of aggressive environmental factors into the interior of the material and the full or partial restoration of the stiffness of the structural element built from this material [1]. In response to the occurrence of material damage, very complex processes of material self-healing are initiated under certain external conditions [2]. According to the definitions developed by RILEM [3], self-healing is any process occurring in a material that involves the recovery and, therefore, improvement of material properties after the occurrence of a destructive action that degraded those material properties.

Over the past two decades, researchers’ efforts have focused on increasing the potential for self-healing of cementitious composites using various techniques and types of additives used in composite manufacturing. The following four major mechanisms of self-healing have been recognized:

- autogenous (natural),
- enhanced by mineral additives including pozzolanic [4,5] and/or hydraulic additives [6,7], expansive agents [8] or crystalline admixtures [9],
- based on microbial activities able to produce calcium carbonate as a by-product of metabolic processes related to nitrogen cycle [10,11,12], aerobic respiration [13], photosynthesis [14] or sulfur cycle [15],
- based on adhesive agents, including single-, double- and multicomponent agents, which can be introduced into cement composite directly [16], in the form of spherical or cylindrical capsules [17] or microtubes constituting simultaneous reinforcement [18], as well as vascular systems [19].

The basic, but not yet fully understood concept, is autogenous self-healing. This type of self-healing is based on the hydration of unreacted cement grains. This is possible if additional ambient water enters the material through the resulting crack and additional space is created for the hydration products to form. In addition, water causes the dissolution of hydration products with relatively higher solubility, such as portlandite, and allows migration of ions from inside the composite structure and redeposition of these products in the cracks. Jacobsen and Sellevold [20] detected that the C–S–H phase, portlandite and ettringite were self-healing products in cracks of high-strength concrete subjected to destructive frost exposure and subsequent treatment in water for three months. Schlangen et al. [21] confirmed the presence of a newly formed C–S–H phase in crack aftercare in water. During the study of Edvardsen [22], calcium carbonate was detected in cracks after autogenous self-healing, which was confirmed in the study by Yang et al. [23]. According to Edvardsen [22], when CO_2_ contained in air dissolves in water, CO_3_^2−^ ions diffuse into the crack. In contrast, calcium carbonate precipitates in cracks when the solution becomes supersaturated. Sisomphon et al. [24] and Parks et al. [25] indicate that calcium carbonate tends to precipitate in cracks near the outer surface. According to the observations of many researchers [26,27], autogenous self-healing also consists of phenomena such as the binding of loose particles of material crushed during crack propagation or debris particles entering the cracks with flowing water. These particles physically fill the cracks reducing the volume needed for filling with self-healing products [3,28].

According to the research, self-healing of cement-based materials at the current levels of advancement is characterized by significant limitations [26], and its effectiveness depends on several key factors determined by the material properties as well as resulting from the service conditions affecting the material. The main material factors are the constituents and proportions of the cementitious composite, primarily the proportion of ingredients actively involved in self-healing processes. In general, the self-healing potential will be the resultant of the quantity of the binder used in the composite, determining the volume of active components in the microstructure and its properties. Due to its commonness, most attention has been paid to the self-healing properties of Portland cement-based composites. In addition to the phase composition of the cement, the degree of grinding is also important [29].

The use of mineral additives, also called supplementary cementitious materials (SCMs), in general, allows for an increase in the self-healing potential as well as an intensification of this process over time [30]. The self-healing rate may decrease when mineral additives are used in large quantities as a substitute for cement, because the pozzolanic reaction is limited by the availability of calcium hydroxide [30]. In such cases, the hydration kinetics of cementitious composites with mineral additives can be low in the first weeks, so in recent years, methods to stimulate and accelerate the self-healing process were proposed: the use of alkaline solutions [5,31], the mixing of different mineral additives [32] including the use of calcareous fly ash [33], lime powder [34] or hydrated lime [35] to increase the calcium content.

The key is the conditions under which the binder hydrates. These conditions are related to the amount of water supplied to the composite during homogenization of the components, the method of placing and finishing of composite, curing procedure, long-term conditions in the place of its use and maintenance during aging. A low ratio of water to binder can lead to a situation where even after a long period of aging, unreacted binder particles will remain in the material. These will constitute a reservoir of binding material that can react and hydrate at a later time [29,36]. Thus, cement-based composites with a lower water-to-binder ratio (w/b) can be expected to have a higher potential for self-healing [37]. However, the potential during crack formation may not be completely utilized as indicated in [28] due to too low permeability of composite matrix, as is the case in high strength concretes, among others. Most of the research in the field of self-healing has focused on materials with high binder content and low w/b. Mor et al. [38] studied self-healing in lightweight, high-strength concretes. The results of Granger et al. [39] confirmed the self-healing of ultra-high-strength concretes with a w/b = 0.2, while no evidence of crack filling was shown in concretes with w/b = 0.35 and 0.48. Subsequent studies on composites using expansive admixtures indicate the possibility of self-healing also in cases of concretes with w/b > 0.4 [40,41].

The factor determining the degree of hydration of cement particles as well as other binders is the age of the composite. Studies on self-healing indicate that this process is most effective in young concretes. Although Zamorowski [42] showed that complete filling of cracks is possible only if they are formed at the initial stage of concrete hardening (during the first 90 h), over several decades, it has been possible to obtain composites in which restoration of the original integrity of the structure is possible many months after the beginning of hydration [28,36], and the effects of autogenous self-healing can be observed even in long-term structures [43].

The type and degree of damage of the material are of key importance for the self-healing process. Zhong and Yao [44] observed that there is a dependence of the intensity of self-healing determined by strength measures on the degree of material damage determined using ultrasonic and strength testing methods. The results of the analyses indicated the existence of a certain threshold of material damage, below which the efficiency of self-healing increases with the degree of damage, while above this threshold, it decreases. This threshold is higher for normal concrete than for high-strength concrete [44].

Because cementitious composites have limited tensile strength, most attention has been paid to the self-healing of cracks. Yang et al. [23], Li and Yang [45] and Yang [46] report that complete sealing is possible for cracks with a width < 50 µm, while partial sealing is observed in cracks up to 150 µm. In similar test conditions [30], it has been noted that full sealing is possible for cracks below a width of 300 µm [32]. Other authors point out that even partial sealing of cracks above 300 µm is possible [24,28]. Therefore, self-healing fiber-reinforced composites are a parallel concept that allows us to control the crack development and to replace single wide cracks with a network of smaller cracks [47].

After the occurrence of damage in the cement composite, the factors that determine the initiation and course of the self-healing process are the environmental conditions. The basic one is the presence of water, which allows the process of cement hydration or migration of various substances deep into the material structure [26]. Water entering the material through cracks in the material allows for further hydration of unreacted cement particles. The possibility of self-healing of cracks in elements exposed to water flowing through cracks has also been confirmed [48]. The course of self-healing was also studied in conditions such as seawater action [49,50].

Another important factor influencing the self-healing process is the CO_2_ concentration in the vicinity of the cement-based material undergoing self-healing. An increase in its concentration allows for intensification of carbonation and accelerates calcium carbonate formation [36]. Carbonation occurs most intensively under cyclically varying conditions, for instance, based on the action of alternating wetting and drying. [23]. To simulate prolonged exposure to different conditions of cement-based composite treatment to intensify the self-healing process, various techniques have been used in research, including increasing the concentrations of key substances in the environment [36] or increasing the temperature and humidity [36,51,52] relative to the natural environment conditioning the dynamics of chemical reactions.

The literature review reveals the complexity of the process of autogenous self-healing of cementitious composites and the factors conditioning its intensity. The degree of complexity is further multiplied by the multitude of combinations of designed material properties of cement-based composites or environmental conditions in which the material is used. This publication shows some of the relationships existing between specific measures of self-sealing effects and the key factors determining this process. These relationships are presented in the example of mortars and concretes with mineral additives: mechanically activated fly ash from the combustion of brown coal in fluidized bed boilers (MAFBC fly ash) and siliceous fly ash from the combustion of hard coal in pulverized coal boilers. So far, the use of fluidized bed combustion ashes as binders [53,54,55], stabilizers in geotechnical work [56,57] or concrete additives [58,59,60] was described in several works, but rarely in terms of enhancing the efficiency of self-healing [37].

## 2. Materials and Methods

### 2.1. The Research Plan

To ensure a research plan covering a broad range of aspects, the study used mortar and concrete samples with different geometries, compositions, material defects and ages at the time of crack formation. It was not possible within this research plan to capture all possible determinants of self-healing, so factors such as water availability, CO_2_ concentration and temperature during care after damage induction were held constant. The key details of the research plan are summarized in Table 1.

The research plan provided for groups of materials differing in age at the time of defect formation, i.e., 14 or 28 days. Mortar samples intended only for compressive strength tests (MT), in which no material defects were induced, constituted a separate group. The research results presented in this publication will be considered within the framework of material types with various w/b (equal to *q*). Details characterizing the materials to be tested and a description of the tests carried out are presented in the following sections.

### 2.2. Preparation of Mortar Samples

The first type of materials were mortars designed to have the same total mass of Portland cement and mineral additives and the same binder-to-aggregate mass ratio. CEN-standard sand, according to PN-EN 196–1, was used as aggregate. The water-to-binder ratio was variable in this case, i.e., 0.3 or 0.5, as well as the degree of Portland cement substitution by mineral additives, i.e., 0%, 5% or 20%. Mechanically activated fly ash from lignite combustion in fluidized bed boilers (MAFBC fly ash) was used as a mineral additive (a commercial product called FLUBET^®^). This product is approved for use as a concrete additive up to 20% by weight of cement [61]. The second mineral additive was siliceous fly ash (class F according to ASTM C618–12a and class V according to PN-EN 197–1) from hard coal combustion in pulverized coal boilers. The characteristics of cement and both mineral additives are presented in Table 2. Details on the phase composition of fly ashes have been given by Tomczak et al. [37].

**Figure 1:**
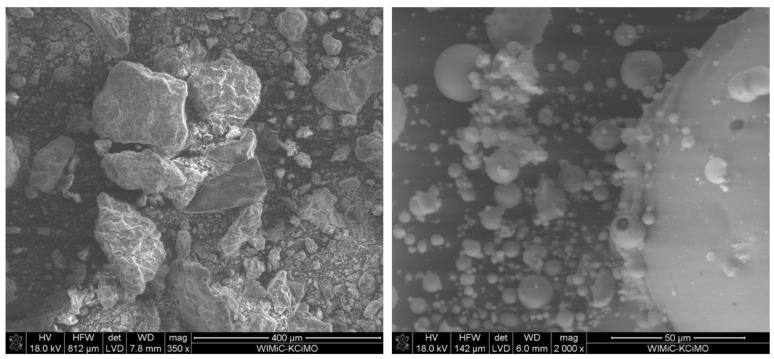
SEM images of grain morphology of MAFBC fly ash (**left**, mag. 350×) and siliceous fly ash (**right**, mag. 2000×).

Morphology of grains of both fly ashes was examined with a scanning electron microscope (SEM) FEI Nova NanoSEM 200. As shown in Figure 1, MAFBC fly ash is characterized by significantly different grain morphology than siliceous fly ash. The developed surface of MAFBC grains, together with high open porosity, contributes to the increased water demand of mixtures with these ashes. However, it has been shown that this type of ash is characterized by significantly higher initial reactivity than siliceous fly ash [37,62].

To ensure comparable workability and air content in fresh mortars, CHRYSO^®^ Optima 185 superplasticizer was used. Additionally, to control crack propagation and geometric invariability in later stages, Astra Polyex Mesh 2000^®^ high-modulus 12 mm long polypropylene fibers (dosage: 2 vol%) were used in the samples intended to study self-sealing effects. The formulations of the designed mortars are shown in Table 3. *Mp(F/S)_q* denotes the mortar with *p*% replacement of cement with MAFBC fly ash (*F*) or siliceous fly ash (*S*) and w/b = *q*.

The samples were formed in tripartite steel molds in two layers and compacted at 1 Hz for a total of 120 s. After 24 h, the samples were unmolded and then deposited in a climate chamber in sealed containers at 20 ± 2 °C and with a relative humidity (RH) above 90% for 13 or 27 days until induction of defects.

### 2.3. Preparation of Concrete Samples

The second material investigated was concrete. The common feature, allowing comparison of the analyzed composites, was an identical volume ratio of cement paste to fine and coarse aggregate, i.e., the active parts of the composite to the inert parts in terms of self-healing capacity. The mix proportions are presented in Table 4. *Cp(F)_q* denotes the concrete mixture with *p*% replacement of MAFBC fly ash cement (*F*) and w/b = *q*.

The variable in this test plan was the water-to-binder ratio, i.e., 0.25 or 0.4, as well as the degree of cement substitution with MAFBC fly ash, i.e., 0%, 5% or 20%. The dosage of superplasticizer was chosen so that the mixtures would have the same consistency. The concrete mixtures were placed in the molds in two equal layers with simultaneous compaction on a vibrating table conforming to PN-EN 12390–2 for a total of 60 s. After demolding, the concrete samples were aged under the same conditions as the mortar samples for 27 days until the induction of defects.

### 2.4. Induction of Defects

Before inducing material defects, and before each subsequent test point, all the specimens were subjected to drying at 30 °C under forced-air conditions in a drying oven for 22 h. The drying was to ensure that the specimens were of comparable low moisture content during testing at all test points. Material defects were performed on half of the scheduled samples, i.e., 3 samples for each mortar and 5 samples for each concrete. The remaining half of the samples were control samples for UPV testing (Section 2.5).

Mortar specimens were subjected to three-point bending to induce cracks with variable widths from 0 to 750 μm under a displacement rate control condition of 1 mm/min and a set limit deformation in the form of deflection of 1.75%. The bend tests were performed using a Zwick–Roell Xforce P high precision machine with a load range up to 20 kN.

Concrete samples used to induce volumetric micro-cracks were subjected three times cyclic to compressive loading up to 80% or 85% and unloading up to 50% of the average uniaxial compressive strength for samples made of mixtures with w/b equal to 0.4 and 0.25, respectively. The compressive strength of the concretes after 28 days of aging was determined on a total of 15 additional samples. The loading and unloading rate of the specimens was 1 MPa/s. The strength testing and crack induction was performed on a servo-controlled testing machine from Walter + Bai AG with a load range up to 3000 kN.

After inducing cracks, the mortar and concrete samples with material defects along with control samples without cracks were stored in water at 20 ± 2 °C in CO_2_-tight containers for up to 122 days.

### 2.5. Test Methods

The research plan included compressive strength tests (MT) on halves of 25 mm × 25 mm × 100 mm rectangular mortar (M) samples made from the mortars without dispersed reinforcement. The objective of this study was to determine the compressive strength as a parameter characterizing the hydration progress of blended binders in time. The mortars were made according to the formulas given in Table 2 but without the use of fibers. Material failure during compression occurs due to crack propagation. Polypropylene fibers with high tensile strength would interfere with the compressive strength determined in this study. Strength tests were performed at 3, 7, 28, 56, 92 and 122 days of curing. The tests were conducted on a Controls Automax 5 testing machine with an attachment suitable for testing 25 mm × 25 mm × 40 mm samples, at a loading rate of 500 N/s.

Mortar samples subjected to cracking were subjected to high-resolution scanning (HRS) to evaluate the changes occurring during curing in the vicinity of the cracks near the outer surface. The side surfaces of the cracked samples were subjected to grinding on a stationary grinder with water removal of the ground material. The grinding was intended to ensure uniformity of the sample surfaces and removal of the cement paste layer to minimize the edge effect of accumulation of newly formed material near the outer surface of the samples. The surface of the samples was digitally captured using an Epson V600 Photo scanner equipped with a CCD sensor with an optical resolution of 6400 dpi and an optical density of D = 3.4. Samples from the group marked *M 28-days* were scanned immediately after crack induction and after 3, 7, 28, 56, 92 and 122 days of subsequent curing. Samples from the group designated *M 14-days* were scanned immediately after crack induction and after 122 days of subsequent curing. The digitally captured images were subjected to computer processing and image analysis using a proprietary method that has been published [63]. This study was complemented by microscopic observations (MO) using a Delta Optical Smart 5MP PRO microscope with digital image capture and a maximum optical zoom of 300×.

**Figure 2:**
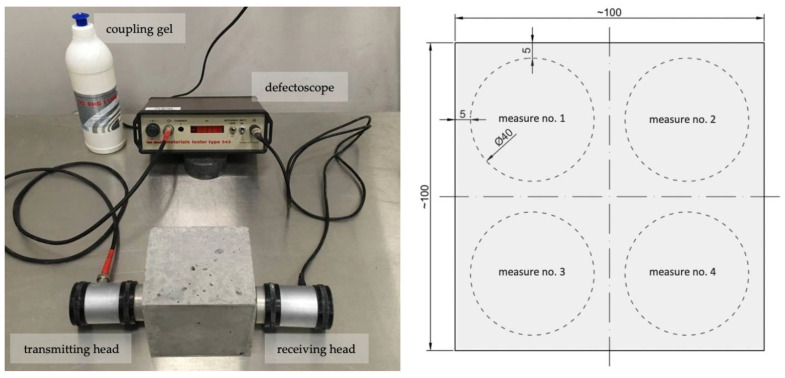
Test stand for ultrasonic wave velocity measurements in concrete specimens and a scheme of transducer head locations on the sample surface (dimensions in mm).

Ultrasonic pulse velocity (UPV) tests were conducted to determine the changes occurring inside mortars and concretes due to material defects and subsequent curing. A Unipan type 543 material tester ultrasonic defectoscope equipped with 40 mm diameter transducer heads was used to study the time of wave passage through material samples (Figure 2).

The transmitting head generated ultrasonic waves with a frequency of 40 kHz. Tests were performed on samples with induced defects and control samples without defects. For samples with defects, ultrasonic wave transit time tests were conducted immediately before and after defect induction and after 122 days of subsequent curing. In control samples, tests were conducted at corresponding time points. For mortar samples, the measurement was made in the direction perpendicular to the plane of the bending forces. The transducer heads were positioned on the samples’ faces so that they did not extend beyond the edges of the specimen. In the case of concrete specimens, measurements were taken in directions perpendicular to the compressive load axis by placing the heads at 4 non-overlapping locations (Figure 2). At each location, the measurement was repeated twice or more until a stable indication of the time the wave passed through the sample was obtained. The obtained UPV data for samples with defects and control samples were processed according to the procedure described in [64], which allows quantifying only the changes occurring in the course of crack self-sealing, i.e., changes in the degree of crack filling, neglecting the changes occurring in the course of progressive hydration of binders.

## 3. Results and Discussions

### 3.1. Microscopic Observations

**Figure 3:**
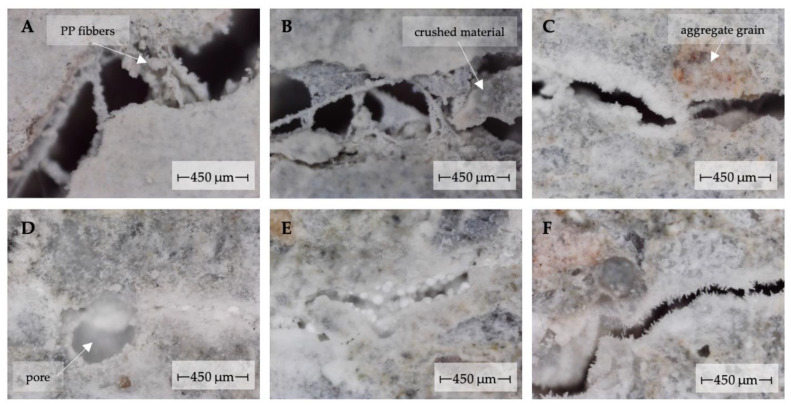
Irregularities in the degree of crack filling by newly formed material in the area of dispersed reinforcement fibers (**A**) or fragments of crushed material (**B**); local irregularities in crack self-sealing in the area of aggregate grains (**C**) and pores (**D**); diversity of morphology of newly formed material in the crack area (**E**,**F**).

The main observation formulated from the microscopic observations concerns the irregularity of the crack self-sealing process in the mortar samples near the outer surface of the material. This observation inherent in all analyzed cases is related to two aspects. The first concerns the irregularity of the local degree of crack filling with the products of the self-sealing process depending on the local crack geometry (regularity of the crack opening distribution, the angle formed by the crack plane to the material surface) and material differences (including the presence of aggregate grains, the material compaction degree and the presence of air pores) (Figure 3).

This irregularity is also influenced by the presence of material debris or contaminants in the cracks formed during crack propagation, as well as by the exposed fibers of dispersed reinforcement, which caused local accumulation of new material in the crack volume.

**Figure 4:**
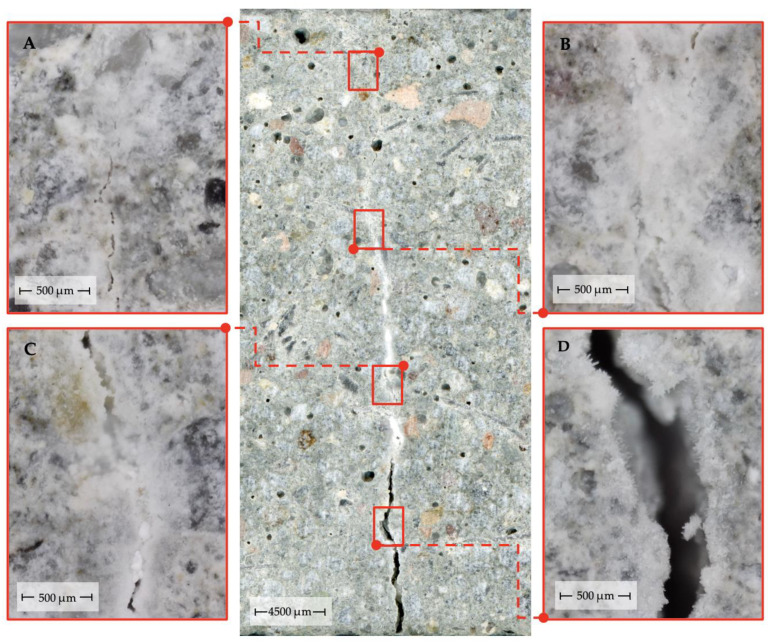
Typical microscopic observations of self-sealing of cracks in mortar samples. (**A**): partial lack of sealing of a crack of <50 μm wide; (**B**): complete sealing of a crack of 100 to 250 μm wide; (**C**): partial sealing of a crack of 250 to 350 μm wide; (**D**): aggregated layers of newly formed material on the surface of a crack of >350 μm wide.

The second aspect is related to the global distribution of the crack width along the crack line at the external material surface, including the maximum crack width in the mortar samples studied (Figure 4).

The course of crack filling with the newly formed material may also differ globally with the SCMs used. It should be noted that the chemical composition of additives used influence the type of self-healing products formed. As cited in Section 1, most commonly, cracks are filled with portlandite, calcium carbonate and C–S–H phase [20,22]. As shown in [37], the use of MAFBC fly ash leads to the formation of new types of self-healing products, such as hydrated calcium aluminates. The formation of such products is stimulated by the high amount of alumina present in MAFBC fly ash [37]. Much of that alumina is reactive [65] and may be a substrate for hydrated compounds formation [66]. Further, the form of silicon oxide present in SCMs plays an essential role. MAFBC fly ash contains a relatively high content of reactive silica (dehydrated clay minerals of disordered structure and high specific surface area)—larger than in case of siliceous fly ashes most commonly used, and thus contributes both to self-healing as well as general strength gain of mortars. In the case of MAFBC fly ash use in cement-based composite, the self-sealing of cracks is characterized by different kinetics compared to composites with siliceous fly ash or with pure cement. The subject of self-healing products and chemical changes due to additives is appealing and indeed worth more attention; however, it requires an adjusted experimental setup. The authors plan to focus on this in the future.

The above observations concerning irregularities in the course of self-sealing, conditioned by local features of the composite in the vicinity of cracks, justify the adopted research methodology. This methodology, based on computer image processing and analysis, allows for crack width measurements at many randomly chosen test points along the crack. These measurements can be repeated at the same real points within the crack after any time based on successive scans of the surface subjected to image registration techniques using scale-invariant processing. Randomly distributed along cracks, numerous and operator-independent measurements of crack width allow one to objectively capture specific changes occurring in cracks during maintenance as a result of self-sealing. The performed study, one of few, allows one to connect the phenomena accompanying self-sealing with geometric parameters of defects.

### 3.2. Microstructure and Age of the Composite at the Time of Defect Formation vs. Self-Sealing Efficiency

**Figure 5:**
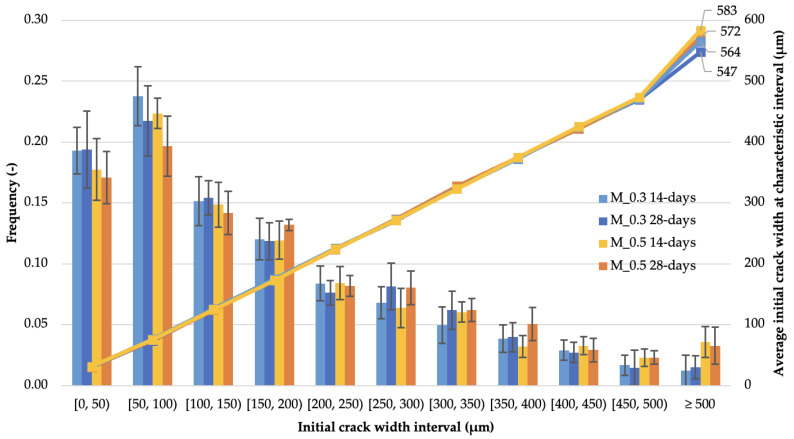
Frequency of crack widths and average initial crack widths analyzed within the width intervals; the intervals ± standard deviation show the variation in crack width frequency within the mortar groups.

High-resolution scans and subsequent analyses were performed on total specimen surfaces of 1200 cm^2^ with a real size of 25 mm × 40 mm. Crack widths were measured at a total of 16,494 points on the material surface immediately after crack induction and after 122 days of curing. The collected datasets for mortars with the same w/b ratios (0.3 or 0.5) and age at crack induction (14 or 28 days) were sorted to the initial crack width into characteristic intervals of 50 μm. The frequency distribution of the cases of the initial crack width, together with the average widths in the characteristic intervals, is shown in Figure 5.

The graphs in Figure 5 show the similar geometrical characteristics of the cracks induced in the groups of mortar samples. The only significant differences are observed between the types of mortars with different w/b and concern the frequency of occurrence and average crack width above 500 μm. Crack width distributions similar for all the considered mortar types enable comparisons of the mortars’ self-healing performance.

As cited in Section 1, the shorter the aging time and the smaller the w/b ratio of the composites, the lower the degree of hydration of the binders used and the more substrates for the chemical reactions accompanying the self-sealing process remain in the composite structure; therefore, composites with a lower degree of binder hydration and a lower w/b ratio are expected to have a higher self-healing potential [30,37]. In the conducted studies, the average change in crack width indicating the total changes occurring in the vicinity of cracks in the course of curing was taken as a measure of self-sealing efficiency. This measure primarily captures the thickness of the newly formed material on the crack walls as a result of self-sealing. To make this measure independent of the initial conditions, the relative change of the crack width to the initial width at a given measurement point was also used. Table 5 shows average values of absolute and relative changes of crack width for mortars and mortar types with the same w/b.

Comparisons of mortar types with different w/b and different ages at the time of defect formation show that the above-mentioned relationships in this section were met. Irrespective of the measurements, mortars with w/b = 0.3, in which cracks were induced after 14 days of aging, showed the highest self-sealing efficiency. The lowest self-sealing effectiveness was observed for the mortars with w/b = 0.5, in which cracks were formed after 28 days of aging. Such correlations can be seen by analyzing the differences in average changes in crack width between individual mortars from different groups and mortar types except for the cases of M20F_0.3, M20F_0.5, M5S_0.3 and M5S_0.5 mortars (Table 5). In the case of absolute changes in the crack opening, the above hypotheses were confirmed in all cases except M0_0.5, M20F_0.3 and M5S_0.3 mortars. Thus, a significant effect on the efficiency of self-sealing was confirmed.

### 3.3. Initial Crack Geometry vs. Cases of Complete Crack Sealing

**Figure 6:**
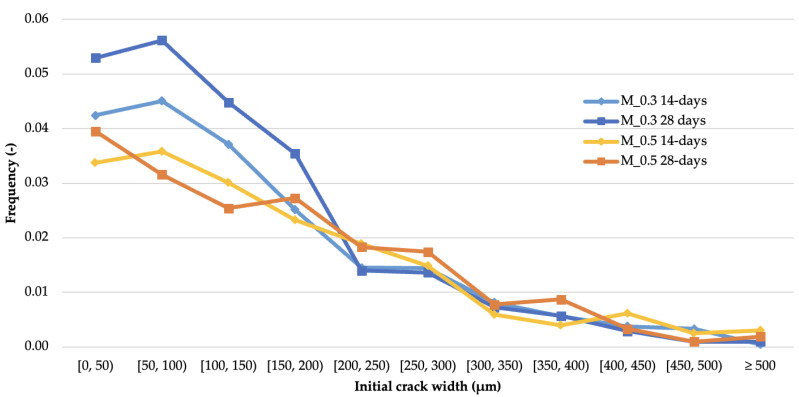
Frequency of full crack closure in ranges of initial crack width after 122 days of self-sealing.

Another measure of self-sealing efficiency is the percentage of cases in which complete filling of crack voids occurred after an assumed time of crack sealing. Figure 6 presents graphs showing the percentage of cases of complete filling of cracks by products after 122 days of curing for each crack width range for different types of mortars. Apart from some deviations from the general course, the plots presented in Figure 6 have a clearly decreasing character with increasing crack width. These data indicate a significant influence of the original crack width on the efficiency of self-sealing, especially when the full integrity of the composite structure is recovered. Due to the much higher number and accuracy of the crack width measurements obtained by the applied test method as compared to the measurements performed manually [23], the results provide additional information not available from earlier studies and new conclusions.

For 3 out of 4 groups of mortars, the maximum recovery of material integrity occurred for cracks of 50 to 100 µm in width. The lower recovery of the narrowest cracks (0 to 50 µm) is consistent with the irregularity of the process shown in Figure 4. The lack of self-sealing effects for these cracks near the outer surface of the material is likely to be the result of a significant reduction of water permeability in this area as the consequence of crack sealing in deeper parts of the material. Similar observations were presented in [64] based on the analysis of material accumulation at the surface of sample fractures. The frequency distributions of full crack closure are very similar among cracks exceeding the width of 200 µm. Significant differences in self-sealing efficiency between the mortar types were observed in the range of cracks of 0 to 200 µm in width. In this range, the highest degree of complete crack sealing was observed for M_0.3 28-days mortars. The highest percentage of complete crack sealing was observed for M_0.3 28-days mortars (24.2% of all measurement points), followed by M_0.3 14-days, M_0.5 28-days and M_0.5 14-days mortars—20.0%, 18.2% and 17.8% of all measurement points, respectively. The largest crack widths, in which complete sealing was observed, belonged to the range of 450 to 500 µm, as well as in isolated cases of crack widths above 500 µm, which is a higher value than that presented in other autogenous self-healing studies for cementitious composites described in [32]. Similar results in this regard have been obtained for studies in self-healing concretes based on bacterial action [67].

In general, the analysis of the results indicates the lack of similarity in the effectiveness of the self-sealing process as assessed by crack width reduction (Section 3.2) and the degree of full crack closure. These results are not inconsistent with each other but different because they are based on differently defined measures of self-sealing. In Section 3.2, it was shown that the accumulation of newly formed material on the crack walls proceeds as expected depending on the w/b ratio and the age of the composite at the time of defect formation. In contrast, the results presented in this section indicate that the complete recovery of cementitious composite integrity is a more complex process. This aspect of the self-sealing process also depends on the initial geometry of the defects.

### 3.4. Binder Activity in the Composite vs. Reduction of Crack Width

**Figure 7:**
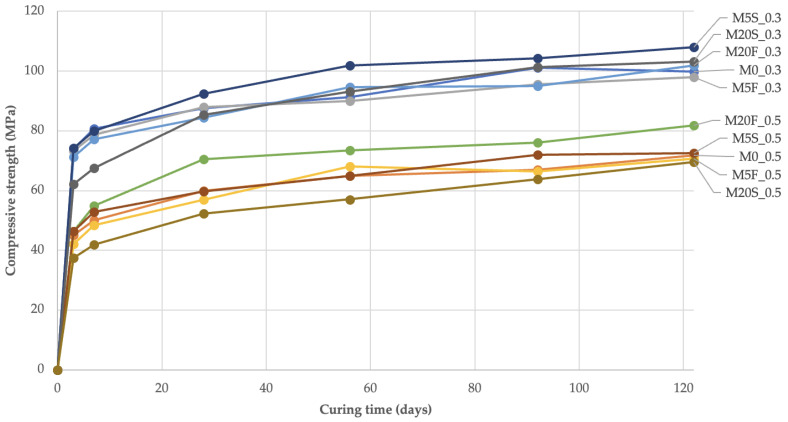
Graphs of changes in compressive strength of mortars over curing time.

The compressive strength of the composite samples in which the respective binders were used was tested as a measure of the activity of the neat cement as well as blended (cement with SCMs) systems. For this purpose, a total of 360 uniaxial compressive strength tests were carried out on halves of 25 mm × 25 mm × 100 mm rectangular samples made from the designed mortars according to the formulations given in Table 2, but without the use of dispersed reinforcement. The test points provided coincided with the test points from the crack width change studies for the *M 28-days* mortars. Compressive strength plots of the mortars over time are shown in Figure 7.

**Figure 8:**
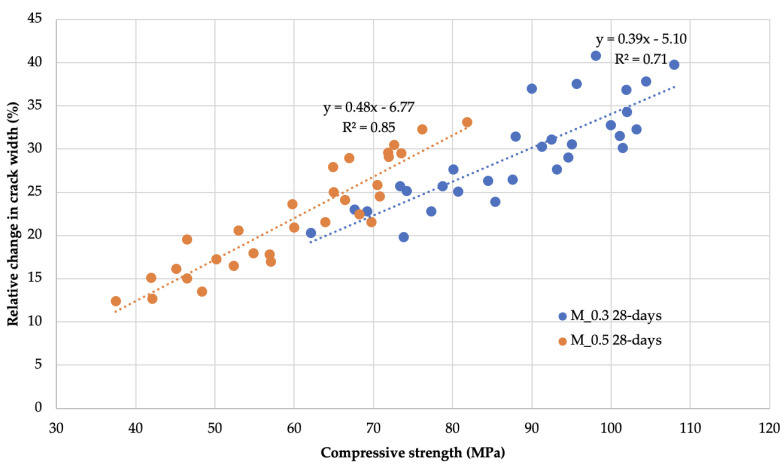
Graph of the dependence of the relative change in crack width after different curing periods on the compressive strength of mortars with cracks induced at day 28 of aging.

The use of cement substitutes in mortars in the form of fly ash has an inconsistent effect on the final compressive strength of mortars. In most cases, after 122 days of curing, mortars with mineral additives showed 1% to 14% higher compressive strength compared to mortars with the same w/b but without mineral additives. Only *M5F_0.3*, *M5F_0.5* and *M20S_0.5* mortars showed decreases in strength in this comparison ranging from 2% to 3%. The highest increases in compressive strength were observed in the first three days of mortar aging with 54% to 75% of the final strength. Thereafter, there was a stable increase in strength until the end of the curing time. Importantly, the specified compressive strengths of the mortars throughout the curing time formed two ranges of values depending on the w/b values. The compressive strength data after 3, 7, 28, 62, 92 and 122 days of curing were compared with the values of one of the visual measures of self-sealing, i.e., the relative change in the crack opening after an equivalent curing time, as shown graphically in Figure 8. The graphs were supplemented with linear regression equations determined by the least-squares method and R^2^ coefficients of determination for each mortar type.

As can be seen in Figure 8, there is a strong and significant linear relationship linking the relative change in crack width to the average compressive strength within w/b ratio groups (R^2^ equal to 0.71 and 0.85). Equally strong are the linearities for mortar groups (R^2^ greater than 0.9) as well as for the entire dataset (R^2^ = 0.79).

As presented above, the study of compressive strength changes in time can be used to predict the composite self-sealing ability and the dynamics of this process. The above considerations also lead to the conclusion that the self-sealing process will be caused by physicochemical reactions accompanying the processes of strength development. On the other hand, the modification of the composition with mineral waste materials leads to significant changes in the composite ability to self-sealing. The presented relationships concern the formation of defects in the mortars after 28 days of aging and the presence of water during the self-sealing time, while all the other cases require investigation.

### 3.5. Degree of Composite Damage vs. Self-Sealing Efficiency

The last considered factor that affects the effectiveness of the self-sealing process is the damage degree of the composite. In this study, UPV was used as an objective measure of the effects of mechanically induced micro- and macro-defects in concrete and mortar samples, respectively, as well as a measure of self-sealing efficiency. Using this quantity, measures of absolute and relative UPV changes were formulated, allowing for the estimation of the magnitude of changes occurring in the microstructure of composites in the course of self-sealing and a comparison of these changes between various composites.

The specific nature of the cement-based composites is that they obtain properties due to a time-stretched hydration of the binders. This means that further natural hydration of the binders may still occur during the self-healing process. Therefore, the relative UPV changes $\Delta v_{t_{i}}$ determined according to Equation (1) were the result of parallel processes of self-sealing and further hydration of the binders. The contribution of this further progressive hydration as a factor causing a change in the values of the physical quantities under study was estimated using measures that are differences of changes occurring in intentionally damaged samples and control samples without defects. In the case of the quantity defined in Equation (2) based on UPV values, the differential values determined in this way provide a measure of changes caused only by the self-sealing process (called pure self-sealing). A more extensive interpretation and application of the $USR_{t_{i}}$ is presented in [64].

(1) $$\Delta v_{t_{i}}=\left({\bar{v}}_{t_{i\;}}-{\bar{v}}_{ind}\right)/{\bar{v}}_{0}\times 100\%$$

(2) $$USR_{t_{i}}=\left(\left(\left({\bar{v}}_{t_{i}}-{\bar{v}}_{ind}\right)-\left({\bar{v}}_{t_{i}}^{ctrl}-{\bar{v}}_{0}^{ctrl}\right)\right)/\left(\;{\bar{v}}_{0}^{ctrl}-{\bar{v}}_{ind}\right)\right)\times 100\%$$

where$\;{\bar{v}}_{t_{i}}$ is the average ultrasonic pulse velocity for a given mortar at time *t_i_*;$\;{\bar{v}}_{ind}$ is the average ultrasonic pulse velocity for a group of samples of a given mortar for the state immediately after cracks were induced; ${\bar{v}}_{0}$ is the average ultrasonic pulse velocity of a given mortar immediately before crack induction at time *t*_0_; ${\bar{v}}_{t_{i}}^{ctrl}$ and ${\bar{v}}_{0}^{ctrl}$ are the average ultrasonic pulse velocities for a group of control samples (without cracks) of a given mortar at the time *t_i_* and *t*_0_, respectively.

**Figure 9:**
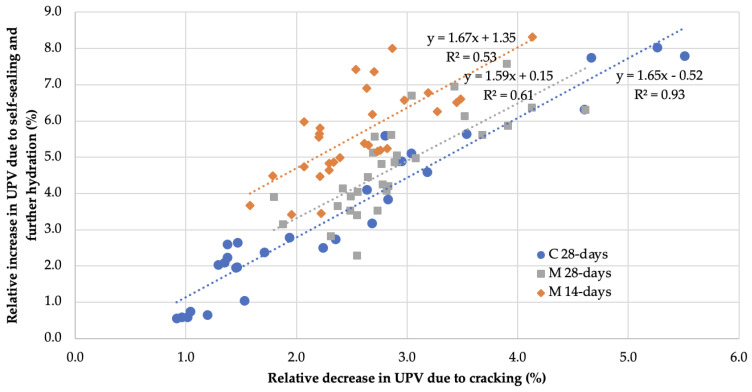
Graph of relative velocity changes of UPV due to self-sealing and further hydration of binders after 122 days of curing versus relative UPV changes due to mechanical induction of defects in concrete and mortar samples.

Figure 9 shows graphs of the relationship of the relative recovery of *UPV* after 122 days of sample curing following the introduction of defects to the relative decrease in UPV caused by the defects created in the mortar and concrete samples. The graphs were supplemented with linear regression equations and R^2^ coefficients of determination for subsets of data describing the phenomena of self-sealing and further hydration of binders in groups of mortars and concretes.

Although defects were introduced differently in mortar and concrete samples, the linear relationships between the measures of UPV change used in all material groups appeared to be best represented by similar directional coefficients. The range of values for the relative decrease in UPV was narrower than for concrete samples subjected to cyclic compressive loading. The obtained values of UPV changes for mortar samples were characterized by a much larger deviation from the fitted trend line than for concrete samples due to the greater variety of recipes provided for testing and the significant differences in self-sealing between the different types. The presence of a linear relationship between the used measures in the form of UPV changes with a positive directional coefficient of the function indicates that the intensity of self-sealing increased in proportion to the degree of damage of the tested materials. Furthermore, the linear relationship over the entire range of data indicates that the damage state introduced in the mortar and concrete samples did not reach the limit beyond which the self-sealing intensity decreases.

In the study of Zhong and Yao [44], the relative change in UPV after peak loading compared to the UPV before loading was a measure characterizing the degree of material failure due to compressive loading. On the other hand, the relative changes in compressive strength determined from strength tests after failure and after a fixed self-healing time were a measure of the self-healing intensity of the normal and high strength concretes tested. The main conclusions of this study regarding the existence of a certain level of specimen damage up to which the intensity of self-healing increases and above which the intensity decreases as the material defect increases, were drawn from the analysis of the relationship between the ultrasonic measure of specimen damage and the strength measure of self-healing. No analysis of the relationship of UPV changes induced by the crack formation and recovery due to self-healing is available in the cited publication; however, tabular raw data of measured ultrasonic wave velocities data were listed [44]. By the processing of these data, evaluation of the relative changes in *UPV* and the respective analysis allowed us to confirm the conclusions presented in this section for the researchers’ data set. Similar to the results in Figure 9, the dependence of the ultrasonic self-healing measure on the ultrasonic damage degree measure of the concretes tested by Zhong and Yao [44] was best described by a linear function. For normal concretes, the directional coefficient of the linear function for the listed relationship takes the value of 2.30 with a coefficient of determination R^2^ equal to 0.90 (six-element sample data), while for high-strength concretes, this coefficient was 1.75 with a coefficient of determination R^2^ equal to 0.74 (nine-element sample data). The differences in the values of the determined coefficients of the directional functions to the value of the coefficient in the analogous relation for the high-strength concretes studied in this paper (equal to 1.65) result, for example, from the different binders used to prepare the concrete mixtures—in the study by Zhong and Yao [44], in addition to Portland cement (ISO 32.5 or 52.5), cement substitutes in the form of class-F fly ash (in normal concrete) or granulated blast-furnace slag and silica fume (in high-strength concrete) were used. Furthermore, in addition to the dimensions of the samples and the type of loading, which were the same for the cited tests and the tests in this paper, the method of defect induction (loading history) had a significant influence on the obtained self-healing results. These results apply only to a portion of the data for samples damaged after 28 days of aging. In the case of the whole datasets, i.e., samples damaged also after 3, 7, 14 or 60 days of aging, the functions describing the linear dependence had different directional coefficients and smaller coefficients of determination. Moreover, the direction of ultrasonic wave transit time measurements was parallel to the plane of compressive load during the induction of defects and not perpendicular, as in the case of the studies carried out in this paper. As a result, the range of relative changes of wave velocity as a measure of damage degree and self-healing intensity was by an order of magnitude larger, which also translated into much larger values of the free expression of linear dependence function.

Based on the analysis of the dependence of the *UPV* recovery magnitude on the material damage degree, different types of results for pure self-sealing quantified by the $USR_{t_{i}}$ values were observed in comparison to the results for general self-sealing. In this case, there was no linear relationship between the quantities describing the UPV changes due to defect formation and pure self-sealing of the material. To quantify the effectiveness of material defect filling depending on the degree of damage in a given material, the Ultrasonic recover-to-damage ratio $URDR_{t_{i}}$ was determined according to Equation (4) based on the ultrasonic self-sealing rate $USR_{t_{i}}$ to the relative change of *UPV* $\Delta v_{ind}$ due to crack induction (Equation (3)).

(3) $$\Delta v_{ind}=\left({\bar{v}}_{0}-{\bar{v}}_{ind}\right)/{\bar{v}}_{0}\times 100\%$$

(4) $$URDR_{t_{i}}=USR_{t_{i}}/\Delta v_{ind}$$

where ${\bar{v}}_{0}$ is the average ultrasonic pulse velocity of a given mortar immediately before crack induction at time *t*_0_; ${\bar{v}}_{ind}$ is the average ultrasonic pulse velocity for a group of samples of a given mortar for the state immediately after cracks were induced; $USR_{t_{i}}$ is the ultrasonic self-sealing ratio at time *t_i_;* $\Delta v_{ind}$ is the average relative decrease in UPV due to crack induction.

**Figure 10:**
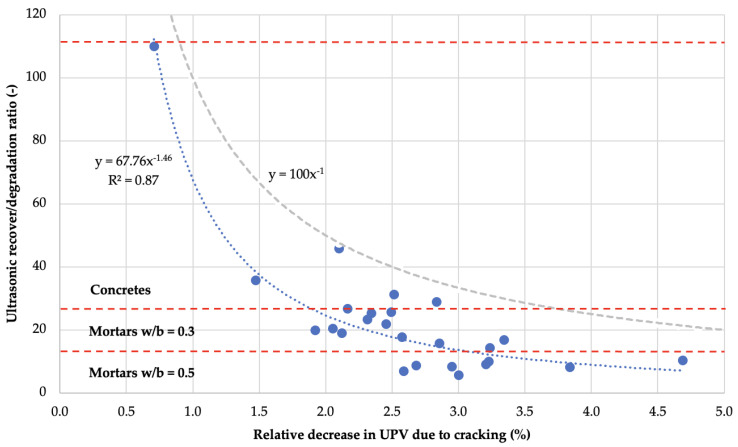
Graph of ultrasonic recover-to-damage ratio due to self-sealing after 122 days of curing versus relative changes in ultrasonic wave velocity due to mechanical induction of defects in concrete and mortar samples. The grey curve indicates the range of possible cases, assuming that the maximum ultrasonic self-sealing ratio is 100%.

The values of $URDR_{t_{i}}$ for all investigated material groups, regardless of the age of the material at the time of defect occurrence, are shown in Figure 10. The position of the point on the scale of $URDR_{t_{i}}$ values clearly depend on the material type. Mortars with w/b = 0.5 had the lowest self-sealing efficiency, followed by mortars with w/b = 0.3, while concretes had the highest. The graph was supplemented with the trend line along with the coefficient of determination R^2^ for the whole dataset. The scatter in position from the trend line is caused by the diversity in composition and/or age at damage induction of the materials. The relationship is impacted by the single far-left point. This indicates that the overall efficiency of self-sealing to composite damage will be the result of many factors, while the adopted index quantifying this dependence is characterized by universality. The graph in Figure 10 also shows the curve $y=100x^{-1}$ (gray dashed line), which refers to cracks completely filled with material and $USR_{t_{i}}=100\%$ thus limits the theoretical range of point locations.

The analysis of the data presented in Figure 10 clearly shows that the phenomena related strictly to self-sealing lead to greater changes in the case of micro-defects in concretes of much smaller widths than in the case of macro-defects in mortar samples. Obviously, the clear division of the ranges of $URDR_{t_{i}}$ values depending on the type of composite indicates the significant influence of the material microstructure on the self-sealing efficiency. Furthermore, the effectiveness of pure self-sealing decreases with the degree of material damage. This confirms the validity of the direction taken by some researchers to modify the deformation parameters of composites to increase their self-healing potential by limiting the crack width formed during damage using mainly dispersed reinforcement fibers with developed strength and deformation parameters.

## 4. Conclusions

An important aspect of shaping self-healing properties of functional materials is the identification of the key factors determining the self-healing potential and, in the case of damage occurrence, also the self-healing effects. In this paper, an attempt was made to quantify the effects of one aspect of autogenous self-healing enhanced by mineral additives, i.e., the ability of cementitious composites to restore the original integrity of the material through spontaneous self-sealing processes. To ensure the diversity of research material, samples of mortars and concretes with different cement substitutes in the form of mineral additives, water-to-binder ratios or age at the time of defects formation and the method of induction of these defects were used. Generalizations of the relationship between specific measures of self-sealing effects and key factors determining this process are presented. The following was concluded:

1. Apart from certain deviations from the general course, the percentage of cases of complete filling of cracks clearly decreased with increasing initial crack width. These data indicate a significant influence of the initial crack width on the efficiency of self-sealing, particularly in terms of the recovery of the complete integrity of the composite structure.
2. In a composite with dispersed reinforcement subjected to mechanical loading, the course of deformation, as well as crack propagation, depends on the deformability of the material and the development of bonds between the reinforcement fibers and the cement matrix. In this case, a shorter aging time was associated with a greater susceptibility to deformation and poorer fiber bonds with the surrounding matrix, which in effect may lead to a change in geometric characteristics of micro- and macro-defects formed during loading in comparison to an analogous material and loading method after a longer aging period.
3. According to the captured data quantitatively describing changes in the vicinity of cracks as a result of post-damage curing, the shorter the composite aging time or lower water-to-binder ratio, the higher the self-sealing effects.
4. As demonstrated by the analysis of the results, the compressive strength as a mechanical measure of binder activity can be used to predict the self-sealing ability of cement composites and the dynamics of this process.
5. The analyses of the dependence of ultrasonic wave velocity changes caused by the created defects and the subsequent self-sealing process confirmed the existence of the dependence of self-sealing efficiency on the damage degree of the material. Moreover, the linear dependence in the whole range of the analyzed data indicates that the damage state introduced in the mortar and concrete samples did not reach the limit above which the intensity of self-sealing decreases.
6. Significant differences in the self-sealing efficiency for mortars and concretes were observed when the results describing pure self-sealing were analyzed. The results indicated that the phenomena related strictly to self-sealing led to more intense changes in the case of micro-defects in concretes, which have much smaller widths than in the case of macro-defects in mortar samples.

## Figures and Tables

**Table 1:** Summary of key information regarding study groups and the research conducted.

Designation of			Material Type	Samples Dimensions (mm)	Sample Count	Crack Indications		Research Conducted			
Groups	Types	Materials				Age (Days)	Method	MT	HRS	UPV	MO
M	*M_q*	*Mp(F/S)_q*	mortar	25 × 25 × 100	360	-	-	+	−	−	−
*M 14-days*			mortar with PP fibers	40 × 40 × 160	60	14	three-point bending	−	+	+	+
*M 28-days*						28					
*C 28-days*	*C_q*	*Cp(F)_q*	concrete	100 × 100 × 100	60	28	cyclic compression	−	−	+	−

Note: in the designation of type or material of samples made of mortar (M) or concrete (C), *p*% means MAFBC fly ash (*F*) or siliceous fly ash (*S*) content and water-to-binder ratio equals to *q*; PP: Astra Polyex Mesh 2000^®^ polypropylene fibers; MT: mechanical testing; HRS: high-resolution scanning; UPV: ultrasonic pulse velocity testing; MO: microscopic observations.

**Table 2:** Chemical composition and physical properties of cement and mineral additives.

	Content of Ingredient (%)														
	SiO_2_	Al_2_O_3_	Fe_2_O_3_	CaO	MgO	TiO_2_	SO_3_	Na_2_O	K_2_O	P_2_O_5_	Cl	Other	Loss on Ignition	CaO_free_ ^1^	Na_2_O_eq._ ^2^
CEM I 42.5R	17.90	5.60	2.61	63.11	1.17	0.36	4.02	0.34	0.78	0.13	0.09	0.25	3.66	1.49	0.85
MAFBC fly ash	37.78	31.55	3.61	10.87	2.00	3.40	3.84	2.13	1.17	0.34	0.03	0.40	2.89	2.65	2.90
Siliceous fly ash	46.92	28.54	7.23	3.39	2.31	1.32	1.87	4.83	2.08	0.31	0.02	0.45	0.72	0.30	6.19

^1^ Based on glycol method according to PN-EN 451–1. ^2^ Na_2_O_eq._ = Na_2_O + 0.658 K_2_O.

**Table 3:** Mortar recipes.

Mortar Type *M_q*	Mortar *Mp(F/S)_q*	CEM I 42.5R (g)	MAFBC Fly Ash (g)	Siliceous Fly Ash (g)	CEN-Standard Sand (g)	Water (g)	SP (g)	PP ^1^ (g)	w/b ^2^ (−)
M_0.3	M0_0.3	550.0	0.0	0.0	1350.0	160.83	5.50	15.63	0.30
	M5F_0.3	522.5	27.5	0.0		156.65	11.00	15.67	
	M20F_0.3	440.0	110.0	0.0		148.30	22.00	15.79	
	M5S_0.3	522.5	0.0	27.5		157.69	9.63	15.70	
	M20S_0.3	440.0	0.0	110.0		149.35	20.63	15.93	
M_0.5	M0_0.5	550.0	0,0	0.0		274.50	0.66	17.61	0.50
	M5F_0.5	522.5	27.5	0.0		273.96	1.38	17.64	
	M20F_0.5	440.0	110.0	0.0		272.91	2.75	17.72	
	M5S_0.5	522.5	0.0	27.5		273.96	1.38	17.68	
	M20S_0.5	440.0	0.0	110.0		272.91	2.75	17.87	

Note: *Mp(F/S)_q* represents a mortar (M) with *p*% MAFBC fly ash (*F*) or siliceous fly ash (*S*) and w/b = *q*. SP: CHRYSO^®^ Optima 185 superplasticizer; PP: Astra Polyex Mesh 2000^®^ polypropylene fibers; w/b: water-to-binder ratio. ^1^ Component not used in mortars provided for compressive strength testing (MT). ^2^ The water contained in the superplasticizer, i.e., 75.9% of the admixture weight, was taken into account in the w/b determination.

**Table 4:** Concrete mix recipes.

Concrete Type *C_q*	Concrete *Cp(F)_q*	CEM I 42.5R (kg)	MAFBC Fly Ash (kg)	Aggregate 0/2 (kg)	Aggregate 2/8 (kg)	Water (kg)	SP (kg)	w/b ^1^ (−)
C_0.25	C0_0.25	480.2	0.0	541.1	1288.4	123.6	10.474	0.25
	C20F_0.25	380.3	95.1			114.6	20.533	
C_0.4	C0_0.4	400.0	0.0			175.3	1.217	0.40
	C5F_0.4	369.4	19.4			170.8	2.205	
	C20F_0.4	314.7	78.7			169.5	4.851	

Note: *Cp(F)_q* represents a concrete (C) with *p*% MAFBC fly ash (*F*) and w/b = *q*. SP: CHRYSO^®^ Optima 185 superplasticizer; w/b: water-to-binder ratio. ^1^ The water contained in the superplasticizer, i.e., 75.9% of the admixture mass, was taken into account in the w/b determination.

**Table 5:** Absolute and relative changes in crack width after 122 days of curing after crack induction; average values are given for mortars and mortar types.

Mortar *Mp(F/S)_q*	Mortar Type *M_q*	Average Crack Width Change (μm)				Average Relative Crack Width Change (%)			
		*M 14-Days*		*M 28-Days*		*M 14-Days*		*M 28-Days*	
M0_0.3	M_0.3	116.9	116.2	80.8	103.9	40.6	37.2	32.2	36.3
M5F_0.3		128.8		118.4		42.3		40.7	
M20F_0.3		110.3		115.4		35.6		36.2	
M5S_0.3		100.6		114.3		29.2		40.2	
M20S_0.3		124.2		90.5		38.2		32.1	
M0_0.5	M_0.5	101.4	110.1	76.9	87.4	31.3	33.3	32.4	28.3
M5F_0.5		113.8		70.7		32.8		23.8	
M20F_0.5		105.6		106.5		33.1		32.5	
M5S_0.5		114.3		90.3		35.7		30.2	
M20S_0.5		115.2		92.5		33.7		22.5	

Note: in the designation of type or material of samples made of mortar *p*% means MAFBC fly ash (*F*) or siliceous fly ash *(S*) content and water-to-binder ratio equals to *q*; in *M 14-days* and *M 28-days* mortars groups designations, the number indicates the age of the mortars at the time the cracks were induced.

## Data Availability

The data presented in this study are available on request from the corresponding author.
